# Validation of the Chemical and Biological Steps Required Implementing an Advanced Multi-Omics Approach for Assessing the Fate and Impact of Contaminants in Lagoon Sediments

**DOI:** 10.3390/metabo14080454

**Published:** 2024-08-17

**Authors:** Anouar Mejait, Aurélie Fildier, Barbara Giroud, Gaëlle Daniele, Laure Wiest, Delphine Raviglione, Jules Kotarba, Eve Toulza, Triana Ramirez, Alexia Lanseman, Camille Clerissi, Emmanuelle Vulliet, Christophe Calvayrac, Marie-Virginie Salvia

**Affiliations:** 1 Centre de Recherches Insulaires et Observatoire de l’Environnement (CRIOBE), 66860 Perpignan, France; anouar.mejait@univ-perp.fr (A.M.); delphine.raviglione@univ-perp.fr (D.R.); jules.kotarba@univ-perp.fr (J.K.); alexia.lanseman@etudiant.univ-perp.fr (A.L.); camille.clerissi@ephe.sorbonne.fr (C.C.); 2 Institut des Sciences Analytiques UMR 5280, Université Claude Bernard Lyon 1, CNRS, 69100 Villeurbanne, France; aurelie.fildier@isa-lyon.fr (A.F.); barbara.giroud@isa-lyon.fr (B.G.); gaelle.daniele@isa-lyon.fr (G.D.); laure.wiest@isa-lyon.fr (L.W.); emmanuelle.vulliet@isa-lyon.fr (E.V.); 3 UFR Sciences Exactes et Expérimentales, Université de Perpignan, 66860 Perpignan, France; 4 Plateau MSXM Bio2Mar, Université de Perpignan, 66860 Perpignan, France; 5 IHPE, Université Montpellier, CNRS, Ifremer, Université de Perpignan Via Domitia, 66860 Perpignan, France; eve.toulza@univ-perp.fr; 6 Laboratoire de Biodiversité et Biotechnologies Microbiennes, LBBM, Sorbonne Université, CNRS, 66650 Banyuls-sur-Mer, France; triana.ramirez@etudiant.univ-perp.fr (T.R.); christophe.calvayrac@univ-perp.fr (C.C.); 7 Biocapteurs-Analyse-Environnement, Université de Perpignan Via Domitia, 66860 Perpignan, France

**Keywords:** contaminants, fate, impact, lagoons, metabolomics, metabarcoding, microbiome, bacterial diversity, sediment

## Abstract

The increasing use of chemicals requires a better understanding of their presence and dynamics in the environment, as well as their impact on ecosystems. The aim of this study was to validate the first steps of an innovative multi-omics approach based on metabolomics and 16S metabarcoding data for analyses of the fate and impact of contaminants in Mediterranean lagoons. Semi-targeted analytical procedures for water and sediment matrices were implemented to assess chemical contamination of the lagoon: forty-six compounds were detected, 28 of which could be quantified in water (between 0.09 and 47.4 ng/L) and sediment (between 0.008 and 26.3 ng/g) samples using the UHPLC-MS/MS instrument. In addition, a non-targeted approach (UHPLC-HRMS) using four different sample preparation protocols based on solid/liquid extractions or an automated pressurized fluid extraction system (EDGE^®^) was carried out to determine the protocol with the best metabolome coverage, efficiency and reproducibility. Solid/liquid extraction using the solvent mixture acetonitrile/methanol (50/50) was evaluated as the best protocol. Microbial diversity in lagoon sediment was also measured after DNA extraction using five commercial extraction kits. Our study showed that the DNeasy PowerSoil Pro Qiagen kit (Promega, USA) was the most suitable for assessing microbial diversity in fresh sediment.

## 1. Introduction

Increased global commercial trade, climate change and the accelerated production of xenobiotics are all factors that increase the risks of environmental contamination, affecting environmental, animal and human health. The increasing use, in number and quantity, of chemical molecules requires improved detection capabilities, a better understanding of their presence, their dynamics in the environment and their overall impact on ecosystems. In fact, it seems essential to set up an integrative transdisciplinary approach to better identify the fate and impact of these contaminants on the microbiome and to investigate the resilience and/or the adaptation capacities of microbial ecosystems. In this context, we have chosen Mediterranean lagoons as a study model. Indeed, Mediterranean lagoons are particular habitats from an ecological point of view, which support the development of remarkable biodiversity, and their productivity is particularly high. As ecosystems at the interface of the continuum “land–lagoon–sea”, they are subjected to chemical and biological pollutants inputs. This human contamination, which can alter the balance of ecosystems, encourages the emergence of pathogenic and/or invasive organisms and is therefore perfectly in line with the issues identified in the “One Health” concept. In the context of this study, the Canet-St Nazaire (France, 66) lagoon constitutes a particularly relevant “model site” since this lagoon seems to present a worrying level of contamination in terms of pesticides, drug residues, alkylphenols, PolyChloroBiphenyls (PCBs) and Polycyclic Aromatic Hydrocarbons (PAHs) [1,2].

The aim of our study was therefore to set up the first steps of a multi-omics methodology for assessing the environmental fate of lagoon organic contaminants, their impact on the bacterial communities and the ecosystem’s response to this exposure (in water and sediment matrices). To date, a great deal of work has been carried out in order to detect and quantify organic pollutants, particularly at trace levels, in water and sediment matrices using chromatographic methods [3,4,5,6,7,8,9]. Only a few recent studies based on metagenomic were developed for evaluating the impact of environmental stresses on the sediment matrix. For instance, Y. Li et al. investigated pollutants’ impact on bacteria in surface water and sediment [10]. C. Fang et al. studied the sediment archaeal and bacterial communities’ responses towards microplastic exposure [11]. On another other hand, metabolomics is currently widely used in fields such as basic biology, medicine, clinical pharmacology, toxicology and nutrition [12,13], but its employment in the environment areas is still limited as it is only just beginning to be used in this field. There are still challenges to be overcome and many developments are needed, particularly regarding data acquisition procedures, i.e., sampling procedures and analytical methods (sample preparation, extract analysis), as well as the analysis of large metabolomics datasets [12,14,15,16], in order to derive maximum benefit from this science and ensure correct and reliable interpretation of the data. Moreover, to our knowledge, no method combining metabolomics and 16S rRNA metagenomic has been developed to address this environmental issue. That is why we propose, in this work, the development of an innovative approach, coupling chemical and microbial methodologies for the evaluation of the fate and impact of organic pollutants in lagoon environments. This new methodology will enable us to obtain correlations between ecological parameters such as microbial diversity and relative abundance and metabolic parameters (biometabolome) that will be useful for identifying biomarkers of environmental exposure to xenobiotics [17]. In this context, this article presents results from analytical chemistry and molecular microbial ecology, enabling us to validate the first steps in the development of a future multi-omics workflow. Firstly, semi-targeted analytical procedures were set up in order to evaluate the lagoons’ contamination status by investigating water and sediment samples. A total of 46 compounds were detected and 28 could be quantified. Among them, pharmaceuticals, pesticides and hormonal steroids were found. In the second stage, a non-targeted metabolomics approach was applied to provide information on the fate of contaminants, specifically targeting the xenometabolome (pollutants and by-products) and their impact on the microbiome by studying the under- or over-expression of endometabolites (matrix metabolites) in the presence of contaminants. To this end, we based our developments on the “Environmental Metabolic Footprinting” (EMF) approach recently set up in the CRIOBE laboratory [18,19,20,21,22,23,24]. Different sample preparation protocols were compared based on the metabolome coverage, the efficiency, and the reproducibility of the extraction method. Understanding microbial diversity in sediment samples becomes of greater importance when investigating the composition and the function of the microbiome. It also provides additional information in terms of dynamics by assessing the effects of the organic pollutants on microbial populations derived from sediment. To determine which environmental DNA extraction commercial kit would offer the most accurate taxonomic view of the sediment microbial ecosystem, this paper also investigates whether the diversity of microbial community structure in lagoon sediments differs substantially depending on the DNA extraction and purification method applied. We therefore evaluated five different DNA extraction kits available on the market and 16S rRNA gene metabarcoding sequencing was performed to calculate alpha diversity metrics.

## 2. Materials and Methods

### 2.1. Sediment and Water Sampling

The water and sediment samples were collected in November 2023 in the “Canet-St Nazaire” lagoon (66, France) at intervals of several meters. Sediment sampling consisted of coring the first 20 cm of sediment. All the sampled sediment was mixed. As the sediment was full of water, it was left for half a day in a beaker to decant. The solid part was recovered and passed through a 2 mm sieve. The sediment was then lyophilized for 24 h (Heto, FD3) for chemical analyses, while the non-lyophilized sediment was used for DNA analyses. The water and sediment samples were stored in a freezer at T = −20 °C until analysis.

### 2.2. Lagoon Status of Contamination—Analytical Development

#### 2.2.1. Chemicals and Materials

- For water sample extraction, we used the following materials:

Ultrapure water was supplied from Fischer and methanol from BioSolve (Dieuse, France). Oasis HLB™ cartridges (6 mL, 150 mg) from Waters^®^ (Guyancourt, France) were used. Labeled internal standards used for extraction (Table S1) were purchased from C.I.L (Ste Foy-La-Grande, France), LGC (Augsburg, Germany), and Sigma (St Quentin Fallavier, France). Their purity was up to 98%. Individual stocks of internal standard solutions were prepared at 250 mg/L in methanol. Standard solutions were stored at −18 °C.

- For sediment sample extraction, we used the following materials:

Ethylenediaminetetraacetic acid disodium salt dehydrate (EDTA) was obtained from VWR Chemicals and QuEChERS salts for Vet Drugs containing 4 g sodium sulfate (Na_2_SO_4_) and 1 g sodium chloride (NaCl) from Agilent Technologies (Les Ulis, France).

- For UHPLC-MS/MS analyses, we used the following materials:

Ultrapure water, methanol, acetonitrile, ammonium formate and formic Acid (FA), UHPLC-MS-grade, were purchased from Biosolve (Dieuse, France). The analytical standards used for quantification (Table S1) were >98% purity and prepared at 1000 mg/L in methanol.

#### 2.2.2. Extraction Protocols

Blank extractions were performed: a blank of ultrapure water and a sample of Fontainebleau sand were extracted and analyzed under the same conditions as the samples.

- Water samples

After filtration on 0.7 μm glass fiber filters with a filtration System (IT30 142 HW) from Millipore (Molsheim, France), 1 mol/L of citric acid solution was added, and 200 µL of a 2 mg/L solution of labeled internal standards was diluted into 250 mL of the sample. Samples were passed through an automated Solid Phase Extraction (SPE) system (AutoTrace™ 280, from Thermo Fisher^®^, Roissy, France) using an Oasis HLB™ cartridge. Next, 250 mL of filtered water sample was loaded. Then, cartridges were rinsed with ultrapure water, dried with nitrogen, and eluted with 5 mL of methanol. Eluates were dried under nitrogen (40 °C) and samples stored at T = −18 °C. Just before injection, samples were suspended in 1 mL of ultrapure water/methanol (90/10, *v*/*v*).

- Sediment samples

For each sediment sample, an aliquot of 2.5 g was weighed in a 50 mL polypropylene centrifuge tube containing one ceramic homogenizer (Agilent technologies) and 100 µL internal mix standard solution at 100 ng/mL was added (list Table S1). The tube was centrifuged at 7000 rpm for 3 min. After 15 min rest, 6 mL EDTA water solution 1 mol/L and 10 mL of acetonitrile were added. The tubes were then transferred to an ultrasonic bath (Branson, 5510 model) for 15 min at 40 Hz, 30 °C. After they were vortexed for a few seconds, a packet of QuEChERS salts was added and the tubes were ground using a Geno/Grinder^®^ (SPEX SamplePrep, Stanmore, UK) 3 min at 1000 rpm. Subsequently, the sample was centrifuged at 6000 rpm for 3 min. Then, 6 mL of the supernatant was transferred to a 12 mL glass tube and evaporated to dryness under a gentle stream of N_2_ at 40 °C. Finally, the extract was dissolved with 600 µL H_2_O/methanol (90/10; *v*/*v*) for the UHPLC-MS/MS analysis. For the “Canet-St Nazaire” lagoon water and sediment samples, 5 replicates were analyzed for each matrix.

#### 2.2.3. UHPLC-MS(/MS) Analyses

A screening of water and sediments extracts was carried out by comparison with databases after injection on a LC-QToF system using an Ultimate 3000 UHPLC system (Thermo Scientific^®^, Waltham, MA, USA) coupled with a quadrupole time-of-flight mass spectrometer (Q-ToF) (Maxis Plus, Bruker Daltonics^®^, Bremen, Germany). Separations were performed using an intensity solo column (2.1 × 100 mm, 1.8 µm particles, Bruker Daltonics^®^) protected with a KrudKatcher Ultra In-Line Filter guard column from Phenomenex (Torrence, CA, USA) maintained at 30 °C; the injection volume was 5 µL. The mobile phases consisted of (A) water/methanol (99/1, *v*/*v*) and (B) methanol with 5 mM ammonium formate and 0.01% formic acid in both phases. The binary elution gradient started with 1% of (B) at a flow rate of 0.2 mL/min for 1 min, gradually increasing to 39% (B) for the next 2 min then increasing to 99.9% (B) at 0.4 mL/min for the following 11 min. The last condition was kept constant for 2 min (flow rate 0.48 mL/min), then the initial conditions (1% (B)–99% (A)) were restored within 0.1 min (flow rate decreased to 0.2 mL/min) to re-equilibrate the column prior to the next injection.

For detection, MS data were acquired using Data-Independent Acquisition (DIA) mode with an electrospray ionization source in positive mode. The mass range was set to 80–1000 Da. The capillary voltage was maintained at 3600 V with a drying temperature of 200 °C. A solution of sodium formate and acetate (10 mM) was used to form clusters for external calibration at the beginning of each run. A quality control of 22 compounds (Table S2) in methanol was injected to correct the database retention times.

Then, most of the substances found by the screening were quantified. To that end, an LC-MS/MS method (Acquity H-Class-Xevo-TQ-S) was developed for these targeted compounds. A liquid chromatographic system Acquity H-Class from Waters (Saint Quentin en Yvelines, France) was used for the compounds separation with a Waters BEH C18 (100 × 2.1 mm, 1.7 µm) column protected with a KrudKatcher Ultra In-Line Filter guard column from Phenomenex (Torrence, CA, USA) with mobile phases composed of (A) ultrapure water with 0.01% formic acid and (B) methanol. The gradient evolves according to the following conditions: 2–100% (B) for 7.5 min, then 3 min maintenance at 100% (B) and finally a return to initial conditions for 3 min. The flow rate was 0.4 mL/min with an oven temperature of 40 °C and the injection volume was 2 µL. A Xevo-TQ-S mass spectrometer from Waters with positive electrospray ionization was used to detect compounds with the following parameters: capillary voltage 2.6 kV; source and desolvation temperature T = 150 °C and 450 °C, respectively; cone gas flow (N_2_) of 150 L/h; desolvation gas flow (N_2_) of 900 L/h and collision gas flow (Ar) of 0.15 mL/min. The quantification was performed in MRM mode; two transitions were followed for each analyte (Table S3). The transition with the highest intensity was used for quantification and the second for confirmation. The retention time and the ratio between the two MRM transitions were followed too to confirm the analytes’ presence.

#### 2.2.4. Data Processing

The screening data processing was performed with Target Analysis for Screening and Quantitation (TASQ)^®^ 1.4 (Bruker Daltonics^®^). All detected signals, couples of exact mass and retention time (*m*/*z*; tR) were compared with two databases: PesticideScreener 2.1 and ToxScreener 2.1 (Bruker Daltonics^®^). These databases contain exact masses, retention time, isotope pattern and fragments of 1200 pesticides and 800 pharmaceutical compounds, respectively.

The quantification was obtained by comparison with internal calibration when labeled internal standards were available or external calibration otherwise.

### 2.3. Omics Approaches Setup

#### 2.3.1. Non-Targeted Metabolomics

- Chemicals and materials

For extractions, acetonitrile of HPLC LC-MS-grade was purchased from VWR International (Fontenay-sous-Bois, France) and methanol of LC-MS-grade was acquired from CARLO ERBA (Val de Reuil, France).

For solid/liquid extractions, 50 mL centrifuge tubes with Polypropylene plug seal caps were bought from Fisher Scientific (Illkirch, France) and 15 mL soda glass test tubes (100 × 16.00 × 0.8–1.0 mm) were purchased from VWR International. Additionally, 0.22 μm Polytetrafluoroethylene (PTFE) filters and 2 mL vials were acquired via Analytic Lab (Castelnau-le-Lez, France). NaCl was obtained from Sigma Aldrich (Saint Quentin Fallavier, France) and MgSO_4_ was purchased from CARLO ERBA.

For EDGE^®^ extractions, EDGE Q-cups, Q-supports, Q-DISC G0 and amber collection bottles were bought from CEM (Saclay, France).

For UHPLC-HRMS analyses, acetonitrile LC/MS, and formic acid LC-MS were purchased from CARLO ERBA.

- Extraction protocols

Four extraction protocols were tested: two based on solid/liquid extractions and two based on an automated pressurized fluid extraction system (EDGE^®^ instrument, CEM Corporation, http://cem.com/edge/, accessed on 2 July 2024). In each case, the acetonitrile solvent and the mixture acetonitrile/methanol (50/50, *v*/*v*) were compared. The four protocols are described below:

(1) QuEChERS extraction: 10 milliliters of water and 15 mL of acetonitrile (LC-MS grade) were added to the 50 mL falcon tube that contained 5 g of lyophilized sediment. The tube was then shaken for 30 s manually and for another 30 s with a vortex device. Salts (1 g NaCl, 4 g MgSO_4_) were then added and the tube was immediately manually shaken for 30 s and swirled on a vortex mixer for 30 s. The tube was centrifuged for 5 min at a rotation speed of 5000 rpm using an Allegra X-30R Centrifuge (Beckman Coulter, Brea, CA, USA). 9 mL of supernatant was recovered and transferred into a 15 mL glass tube and evaporated to dryness under vacuum at T = 30 °C (EZ-2plus evaporator, Genevac, Ipswich, UK). The dry residue was redissolved in 1 mL of acetonitrile. The extract was passed through a 0.22 μm PTFE filter to be transferred in a HPLC vial (with insert).

(2) Solid/liquid extraction using acetonitrile/methanol (50/50, *v*/*v*) mixture: 15 mL of a mixture of acetonitrile/methanol (50/50, *v*/*v*) (LC-MS grade) was added to the tube that contained 5 g of lyophilized sediment. The tube was then shaken 30 s manually and 30 s with a vortex device. The tube was centrifuged for 5 min at a rotation speed of 5000 rpm using an Allegra X-30R Centrifuge (Beckman Coulter, Brea, CA, USA). After that, 12 mL of supernatant was recovered and transferred into a 15 mL glass tube and evaporated to dryness under a vacuum at T = 30 °C (EZ-2plus evaporator, Genevac, Ipswich, UK). The dry residue was redissolved in 1 mL of a mixture of acetonitrile/methanol (50/50). The extract was passed through a 0.22 μm PTFE filter to be transferred in a HPLC vial (with insert).

(3) EDGE^®^ acetonitrile: 5 g of lyophilized sediment sample was packed in the EDGE Q-cup™ with a G0 filter at the bottom. The extraction was performed at T = 45 °C in two cycles. For cycle 1, 10 mL of acetonitrile was passed through the sample from the top and 10 mL from the bottom. A rinsing was performed between the 2 cycles (5 mL of acetonitrile). For cycle 2, 10 mL of acetonitrile was passed through the sample from the top. A final rinsing of the instrument was performed (15 mL acetonitrile).

(4) EDGE^®^ acetonitrile/methanol (50/50): the extraction steps are the same as described above with the mixture acetonitrile/methanol (50/50) as the extraction solvent. Also, the dry extract obtained at the end was redissolved in 1 mL of the same mixture, i.e., acetonitrile/methanol (50/50).

For all the protocols, extractions were performed without sediment matrix and these samples were called “extraction blank”. This allows the identification of compounds coming from the extraction systems (solvents, 15 mL falcon tubes, pipette tips, filters, EDGE^®^ instrument) and therefore allows us to select only endo- and xenometabolites coming from the sediment matrix. The extracts were kept in a freezer (T = −20 °C) prior to analysis.

- UHPLC-HRMS analyses

UHPLC-HRMS data acquisition was carried out using a Vanquish UHPLC system connected to a high-resolution mass spectrometer (Q-Exactive-Plus, Thermo Fisher, Thermo Fisher Scientific, Waltham, MA, USA). Compounds were analyzed using a Luna Omega Polar C18 column (particle size: 1.6 μm, pore size: 100 Å, length: 100 mm, internal diameter: 2.1 mm, solid support: fully porous silica) fitted with a pre-column (AJ0-9532 polar C-18, 2.1 mm) from Phenomenex (Torrance, CA, USA). This kind of chromatographic column was chosen rather than a conventional C18 column, as it allows a better retention of polar and semi-polar compounds and hence permits us to broaden the range of polarity of the metabolites analyzed. The mobile phases consisted of two solvents: (A) H_2_0 with 0.1% formic acid, and (B) acetonitrile containing 0.1% formic acid. Separation was carried out using the following elution gradient: 0% (B) for 2.5 min, then solvent (B) increased from 0 to 100% between 2.5 min and 15 min, remained constant at 100% (B) between 15 and 17 min, then returned to initial conditions, i.e., 0% (B) in 1 min and remained under these conditions for 2 min, i.e., until 20 min. The chromatographic column temperature was maintained at T = 30 °C in a column oven. The injection volume was set to 5 μL and the mobile phase flow rate was set to 0.400 mL/min.

For detection, MS data were acquired using an ESI electrospray ionization source in Switch mode (simultaneous acquisition in positive (ESI+) and negative (ESI-) modes). Also, MS/MS data were acquired using the Data-Dependent Acquisition (DDA) mode. For MS/MS, ESI- and ESI+ data were acquired separately. The scan range was set from *m*/*z* 90 to 1000. The capillary voltage was maintained at 3200 V and −3200 V for ESI+ and ESI-, respectively; the capillary temperature was set to T = 320 °C.

- Data processing

The pre-processing of the MS data obtained in UHPLC-HRMS was performed on WorkFlow4Metabolomics platform. Firstly, a “CentWave peak picking” step was carried out. It permitted us to extract the ions from each sample and to integrate them by filtering them according to defined parameters (ROIs were considered when detecting 5 consecutive scans with minimum intensity equal to 1 × 105 and 5 × 105 for ESI- and ESI+ data, respectively; maximum *m*/*z* deviation of 10 ppm; S/N cutoff: 10). After merging all the data together, a “PeakDensity grouping” (bandwidth = 20 s) was performed in order to group the different peaks corresponding to the same analyte between samples (different extraction protocols tested, 5 replicates per modality + “extraction blanks”, 3 replicates). Then, a loess retention time adjustment was applied (degree of smoothing = 0.8) followed by another grouping (bandwidth = 15 s). The next step is “fillChromPeaks”, which performs any additional integration for each variable. Indeed, this function attempts to find signals that may have been missed during the first peak-picking step.

The data were grouped by sample type (sediment samples vs. extraction blank samples). We processed a generic filter on the DataMatrix obtained as follows: features must be present in less than 2 blank extraction samples and in at least 4 sediment samples. This means that only features that are representative to sediment endometabolites and xenometabolites are kept and features coming from the extraction system are removed.

The data matrix obtained after the pre-processing detailed above was statistically processed on MetaboAnalyst platform. An OPLSDA was performed comparing sediment samples and extraction blanks and the VIPs > 1.5 were selected (on the first component, separating the 2 modalities sediment vs. blank); this permitted us to select the most important sediment metabolites. Also, it corresponded to a second filtering of blank contaminants ensuring that only metabolites from sediment would be selected. A total of 883 features were obtained (578 and 305 features in ESI- and ESI+, respectively). A log transformation was performed for the Heatmap analysis.

As for the putative identification, MS and MS/MS data were processed using Compound Discoverer (Thermo Fisher Scientific-ChemSpider, mzcloud, Metabolika databases) and SIRIUS software. Level 3 (putative characterized compound class) and level 2 (putative annotated compound) identifications were performed. These putative identifications were checked based on (1) the coherence with the sediment matrix studied; (2) the substance chemical properties (coherence between the substance logKow and its retention time) and the different software scores based on the exact mass, the isotopic pattern and the MS/MS fragmentation.

#### 2.3.2. DNA Extraction and Metabarcoding Analyses

- Protocols for extracting DNA from sediments and assessing DNA quantity and quality

A literature search was undertaken regarding the extraction of DNA from sediments to determine the most efficient kit in terms of DNA quantity and quality [25,26,27,28]. Five commercial kits with the best results in both soil and sediment matrices were selected. These kits were the Quick-DNA Fecal/Soil Microbe kit (Zymo Research, Irvine, CA, USA), DNeasy PowerSoil Pro Qiagen kit (Promega, Madison, WI, USA), FastDNA Spin Kit for Soil and Magbeads FastDNA kit for Soil (MP Biodemicals, East Granby, CT, USA) and E.Z.N.A Soil DNA kit (Omega Biotek, Norcross, GA, USA). Extractions were carried out on fresh sediments and according to each kit’s protocol. Mechanical lysis was performed using the Mikro Dismembrator S^®^ (Sartorius Group, Göttingen, Germany) at 3000 rpm for 5 min. The quantity and quality of extracted DNA was obtained using the Epoch Biotek (Thermo Fisher Scientific, Waltham, MA, USA) and the Gen5 software. In addition, the integrity of genomic DNA was determined by visualizing approximately 200 ng of DNA on a 1% agarose gel (*w*/*v*) containing 0.25 μg/μL of ethidium bromide (EtBr), run in 1 × Tris-EDTA buffer at 100 V.

To assess DNA quality and check the presence/absence of PCR inhibitors, the 16S ribosomal DNA gene was amplified using universal primers 27f (5′-AGA GTT TGA TCM TGG CTC AG-3′) and 1492r (5′-TAC GGY TAC CTT GTT ACG ACT T-3′) [29]. Amplification reactions were carried out in a reaction volume containing 200 μM dNTP (Promega, USA), 2 mM MgCl2 (Promega, USA), 1.25 U GoTaq DNA polymerase (Promega, USA), 1 × GoTaq polymerase buffer (Promega, USA), 0.5 μM of each primer (Microsynth, Balgach, Switzerland) and ultrapure water (Promega, USA). The PCR reaction was performed using a T100 Thermal Cycler (Bio-Rad, Hercules, CA, USA) with the following amplification program: 35 cycles of 5 min at 95 °C, 45 s at 92 °C, 45 s at 52 °C, 45 s at 72 °C and a final cycle of 2 min at 72 °C. To check if PCR amplification had worked correctly, 12 μL of amplicons were loaded and separated by electrophoresis on 1% agarose gel stained with GelRed solution (Nucleic Acid Gel Stain, Biotum), placed at 100 V for 25 min in 1 × TBE solution. The 100 bp DNA Ladder molecular size marker (BioLabs, Ipswich, UK) was used, and the gel was revealed using the DOC-PRINT-VX5 stand-alone gel imager (Vilbert Lourmat™) and photographed using an ECX-20.M transilluminator (Vilbert Lourmat™).

- Metabarcoding of 16S rRNAgene

Next, 16S rRNA amplicons libraries were prepared from DNA extracts also with a two-step PCR protocol targeting the hypervariable V3-V4 region with the 341F (5′-CCTACGGGNGGCWGCAG-3′) and 805R (5′-GACTACHVGGGTATCTAATCC-3′) primer pair [30]. Sequencing was performed by the Bio-Environment platform (University of Perpignan Via Domitia, France) using a Miseq illumina sequencing kit v2 in a 2 × 250 bp paired-end.

- Bioinformatic analysis of amplicons sequences

The DADA2 package [31] (truncLen = c(230,220); maxN = 0; maxEE = c(2,2); truncQ = 2) was used to define amplicon sequence variants (ASV). We then used the Silva database (Version 138, 27 March 2020) to compute the taxonomic affiliations. The dataset was filtered for singletons, and we have eliminated all the ASV where the genus was not defined (=NA). Rarefaction curves were computed using the {phyloseq}, R package (version 1.38.0) and the ggrare function [32]. The rarefy_even_depth function was used to subsample datasets; the estimate_richness function was used to compute alpha diversity metrics (Chao1, evenness, Shannon, and Pielou). The PCoA analysis was performed using the stats package (version 4.3.1) and Vegan package (version 2.6.4) [33]. All the graphs were generated using the ggplot package (version 3.4.3) [34] and Venn diagram package (version 1.7.3) [35].

## 3. Results and Discussion

### 3.1. Lagoon Status of Chemical Contamination

Semi-targeted analytical methodologies were developed for the analysis of both water and sediment matrices from the “Canet-St Nazaire” lagoon. These developments were carried out based on previous works performed at the Analytical Sciences Institute (ISA, Villeurbanne, France) [36,37]. Rigorous sample preparations were set up in order to obtain reproducible and sensitive analytical methods. Water samples were extracted by SPE (Auto-Trace 280 Thermofisher) on Oasis HLB cartridges while sediment samples were extracted with 10 mL acetonitrile using the QuEChERS method with NaCl salts. On the one hand, a screening was carried out by comparison with databases, after injection on an LC-QToF system (Ultimate 3000-Bruker Maxis Plus); it allowed the detection of 46 substances belonging to pharmaceuticals (psycholeptics, neuroleptics, antidepressant, analgesics, antiarrhythmics, hypertensives, antihistamines/antiallergics, antibiotics and antimycotics), pesticides (herbicides, fungicides and insecticides) and hormonal steroids (progesterone and testosterone). Among these substances, 28 were quantified on an LC-MS/MS system (Acquity H-Class-Xevo-TQ-S). A triple-quadrupole mass spectrometer was used for the quantification as it is a sensitive and selective instrument and therefore seems to be the most adapted for xenobiotic quantification in complex environmental matrices (waters and sediments). The results of the quantification are displayed in Table 1 for water and sediment samples, respectively. Substances were classified in this Table 1 by compound type (pharmaceuticals, pesticides and hormonal steroids) and sorted from the most concentrated in water to the least concentrated.

A very low Method Limit of Quantification (MLQ) was obtained, between 0.02 and 1.2 ng/L for the water matrix and between 0.001 and 0.1 ng/g for the sediment matrix. The 28 quantified compounds were found at concentrations between 0.09 and 47.4 ng/L and between 0.008 and 26.3 ng/g for water and sediment samples, respectively. For each matrix (water, sediment), five analytical replicates were performed that allowed us to calculate the coefficients of variation (CVs, %). For water samples, the CVs are very low, as many are inferior to 10%. Only Atrazine has a CV > 30%, but it is explained by a different value for one replicate. This could be due to the fact that water was taken from different points located a few meters apart. For the sediment matrix, CVs are higher and could be explained by the lower concentrations found in this compartment. For water samples, it can be observed that some pharmaceuticals (Flecainide, Oxazepam and Carbamazepine) are found in high concentrations. Also, some pesticides and their by-products are highly prevalent in the lagoon such as Terbuthylazine and particularly its by-products as well as Carbendazim although banned for agricultural use in the EU since 31/12/2009 [38]. As for hormonal steroids, they are present in relatively low quantities. Most of the compounds found in the water were also detected in the sediment matrix, but at lower concentrations. These analyses regarding the lagoon’s status of contamination confirm previous observations [1,2]; indeed, there is a worrying level of xenobiotic contamination. It is hence worth evaluating their fate and impact on the lagoon ecosystem. These results regarding the lagoon’s contamination status will enable us to select relevant organic contaminants, in terms of their presence in both environmental matrices (water and sediment), their quantity and their association with different kind/families of contaminants, as model molecules for our future metabolomics studies under controlled conditions using instrumented sediment bioreactors.

### 3.2. Omics Approaches Setup

#### 3.2.1. Non-Targeted Metabolomics

The goal of the non-targeted metabolomics study was to find the optimal procedure for analyzing the major part of the sediment meta-metabolome, which includes both the xenometabolome (contaminants and by-products) and the microbial metabolome, while being reproducible. We aimed to extract as much information as possible from the meta-metabolome. Several studies were already reported in order to analyze pollutants in sediment matrix. Different kinds of extraction have been used, such as the Soxhlet method [3], ultrasound and microwave-based methodologies [4,5,6,7] and the ASE (Accelerated Solvent extraction) technology [8,9]. Also, QuEChERS extraction, based on the use of acetonitrile, is often used for the environmental matrix [37,38,39,40,41,42,43,44,45,46,47]. The Sohxlet methodology is known to be reproducible and efficient, but its main drawback is that it is time consuming and therefore is not adapted for metabolomics for which a large number of samples need to be analyzed. The ASE technique is now used. It is rapid, efficient and automated and de facto reproducible. As for the extraction solvents, water, methanol and acetonitrile alone and in mixtures were employed [3,4,5,6,7,8,9]. Preliminary tests were carried out with water-containing extraction solvents (acetonitrile/methanol/water), and they conducted to extracts with a high salt content due to the high quantity of salt in the sediment matrix, which can pose detection problems in mass spectrometry; water was no longer used.

Based on these findings, we chose to compare a solid/liquid extraction including the QuEChERS extraction, which was easy to set up with an automated extraction. A new instrument was investigated: the Energized Dispersive Guided Extraction (EDGE^®^) automated system, which was introduced recently by CEM Corporation (CEM Corporation, http://cem.com/edge/, accessed on 2 July 2024 [48]) based on the same ASE principle. Four extraction methodologies were therefore tested and ranked. Two solid/liquid extraction methods were evaluated: the QuEChERS method and an extraction based on the use of the mixture of acetonitrile/methanol (50/50). A mixture of acetonitrile and methanol as extraction solvent was also evaluated in an attempt to extend the polarity range of the extracted metabolites. This could be useful for an untargeted metabolomic approach, as it could allow the analysis of a variety of metabolites. On the other hand, the 100% acetonitrile and acetonitrile/methanol (50/50, *v*/*v*) solvents were also tested with the EDGE instrument, allowing fast and automated extractions, at a temperature T = +45 °C. A higher temperature (T = +100 °C) was also investigated in order to improve the extraction efficiency thanks to the use of both high temperature and high pressure. However, a loss of metabolites was observed at this high temperature compared to the temperature of T = 45 °C. The lower temperature was therefore chosen, which is in agreement with the procedure recommended by CEM for soil matrices (CEM Corporation, http://cem.com/en/literature?application=Extraction&literature_type=Application_Notes). The comparison of the four extraction methods was performed in terms of sediment meta-metabolome coverage, procedure efficiency and reproducibility.

- Sediment meta-metabolome coverage and method efficiency

**Figure 1:**
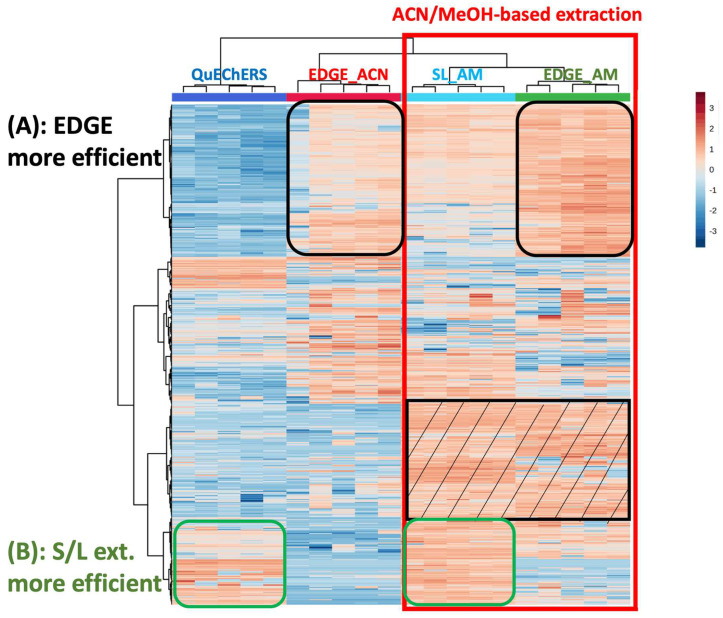
Heatmap analysis (characteristics in rows, sample types/extraction conditions in columns, color intensity related to metabolite concentration -red present and blue absent) of the comparison of extraction protocols in terms of sediment metabolic profiles; (**A**) corresponding to EDGE^®^ extraction and (**B**) corresponding to solvent/liquid (S/L) extraction.

The sediment meta-metabolome coverage was investigated using a Heatmap. It is a 3D representation that enables visualization of the intensity of the features (rows) in the different samples (columns). The different samples, i.e., the different extraction conditions, could therefore be compared. Hierarchical clustering can also be performed; this allows for feature aggregation, due to the correlation of their intensities throughout the samples, as well as samples aggregation, which is explained by similarities in their metabolic profiles. The Heatmap displayed in Figure 1 was performed on the VIP > 1.5 selected from the comparison between blank extraction samples and sediment samples, as explained in part 3.1.4) regarding the metabolomics data processing. These features correspond to the sediment metabolites. The processing of these data led to the selection of 883 features in total (578 and 305 features in ESI- and ESI+, respectively). A log transformation was performed on the data to decrease the size effect and to avoid losing metabolites with low intensity. Moreover, this helped to convert a skewed distribution of the metabolomics data into a Gaussian distribution. Hierarchical clustering (Ward algorithm) was performed on both features and samples (extraction protocols). Firstly, it could be observed that the type of extraction was clustered (due to similarities in their metabolic profiles). Extractions using acetonitrile (ACN) were grouped together on the left, while the extractions with the acetonitrile/methanol mixture (AM) were clustered on the right. We clearly observed that the AM solvent permitted more compounds to be extracted compared to ACN alone (red square), with the better-extracted metabolites being evidenced in the black striped square. The use of a mixture of solvents was likely to extract compounds belonging to a wider polarity range, as expected. On the other hand, it was observed that some metabolites were better extracted with the protocols using the EDGE^®^ system (Figure 1A), which could be explained using higher temperature. However, the solid/liquid methods were better for extracting other compounds (Figure 1B). One hypothesis to explain this result is that these metabolites may be more sensitive to the matrix effect that could occur to a greater extent with EDGE^®^ methods compared to solid/liquid protocols. The EDGE^®^ method may extract more interferents from the matrix that could interfere with the analysis of these compounds by mass spectrometry (Figure 1B). Another explanation is that these metabolites could be degraded with EDGE^®^ using a higher temperature.

**Figure 2:**
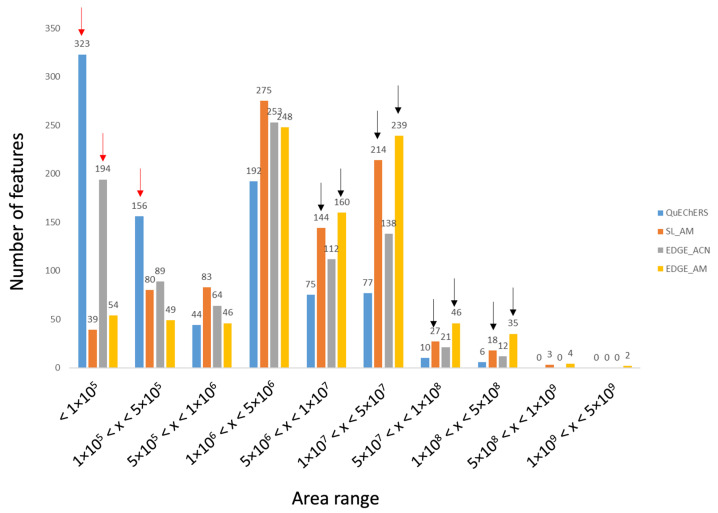
Distribution of the number of features versus peak area ranges representative of the amount of substance present in the extract.

To better reflect extraction efficiencies, the number of features vs. increasing peak area ranges were represented for the four tested protocols and are displayed in Figure 2. This representation is in accordance with the Heatmap, i.e., acetonitrile/methanol protocols are more efficient compared to acetonitrile-based extractions. Indeed, more compounds have low areas with ACN methods (red arrows) whereas more metabolites have high areas for AM procedures (black arrows). Also, it can be observed that for the AM solvent, the EDGE^®^ protocol seems slightly more efficient than the solid/liquid one (yellow vs. orange).

We concluded that the acetonitrile/methanol solvent provides better coverage of the sediment meta-metabolome, with EDGE protocols providing a slight gain in efficiency over the solid/liquid method. However, the reproducibility of the method needs to be assessed, as this is a key factor in metabolomics.

- Method reproducibility

**Figure 3:**
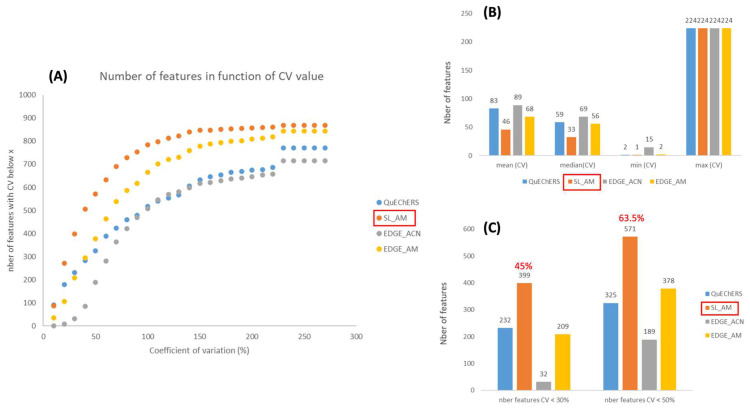
Evaluation of the reproducibility of the 4 extraction protocols tested. (**A**) CV distribution for the 883 selected features; (**B**) CV mean, median, minimum and maximum; (**C**) number of features having a CV < 30% and <50%.

In metabolomics, reproducibility is really crucial when it comes to assessments, as a large number of samples are analyzed in a single study. In order to evaluate this, the coefficients of variation (CVs) were calculated for each feature in the different modalities, i.e., for the diverse extraction protocols. Figure 3A shows that the AM-based extractions (orange and yellow curves) are more reproducible compared to the protocols using only ACN (blue and gray curves), as more features have low CVs. This observation is confirmed by the plots in Figure 3B,C, which compare the mean, median, minimum and maximum CVs, as well as the number of features with a CV < 30% (analytical threshold) and 50% (accepted threshold in metabolomics [49]). In the previous section, we observed that acetonitrile/methanol-based extraction methods were more efficient than those based on acetonitrile, as they showed higher peak areas for the selected features. An inverse relationship was observed between CVs and peak areas, which was to be expected, since we can assume larger errors on small chromatographic peaks, close to the noise level. On the other hand, if we focus on the AM protocols, Figure 3 also shows that solid/liquid extraction is more reproducible than that using the EDGE instrument. Thus, 45% and 63.5% of the features have CVs below 30% and 50%, respectively, for the solid/liquid procedure using AM as the extraction solvent (Figure 3C). This is surprising, as we might have thought that EDGE^®^, being an automated system, would have been more reproducible. However, the EDGE^®^ system is suitable for matrices that are reduced to a fine, highly homogeneous powder for packing into Q-cup EGDE (extraction cell) (CEM Corporation, http://cem.com/en/literature?application=Extraction&literature_type=Application_Notes, accessed on 1 August 2024). The freeze-dried sediment was really “compact” and perhaps not homogeneous enough in the Q-cup. On the other hand, the tests were carried out with 5 g of freeze-dried sediment, because for the following stages, which will be carried out in bioreactors, we will only have 5 g of freeze-dried sediment. This is the minimum quantity of sample to be placed in the Q-cup according to CEM recommendations. However, it may not be enough; it would not fill the Q-cup sufficiently. In the case of solid/liquid extraction, we shake and vortex, so the solvent can probably penetrate the sample matrix more evenly. At this stage, we therefore conclude that the EDGE^®^ system may not be suitable for the sediment matrix or that the sample preparation needs to be reviewed.

For the metabolomics part, we compared a conventional solid/liquid extraction method with an automated extraction system (EDGE^®^) likely to offer superior repeatability performance, a major criterion in our study. Unfortunately, EDGE^®^ did not provide good repeatability. We therefore validated solid/liquid extraction using acetonitrile/methanol as the most suitable protocol for our experimental conditions. As we have shown, in addition to its analytical reproducibility, this method is also efficient and covers a large part of the sediment meta-metabolome. In addition, the solid/liquid methodology is less expensive and more environmentally friendly than EDGE^®^, as it uses less solvent (15 mL compared with 40 mL).

- Putative family/metabolites identification

Some putative family/metabolites identification was carried out using Compound Discoverer and Sirius software (see Section 2.3.1) to process the metabolomics data. In this section, we focus only on the endometabolome, as a detailed study of the xenometabolites present in the lagoon (water and sediments matrices) was already performed (see Section 2.2). In terms of endometabolites, benzoic acid, nucleosides, fatty acyls, lipids, and peptides were found. Table 2 displays the various identifications. EIC chromatograms for some of these metabolites and obtained with the chosen extraction protocol (solid/liquid extraction using acetonitrile/methanol) are displayed in Figures S1 and S2. Lastly, since we have also observed that the endometabolites found are predominantly semi-polar and apolar, and to benefit from a more integrative approach, in future work, we plan to develop methods for analyzing polar compounds via HILIC column in UHPLC-HRMS (Hydrophilic interaction chromatography) as well as using NMR analyses.

#### 3.2.2. Effects of DNA Extraction Methods on the Determination of Soil Microbial Diversity in Sediment

- DNA extraction and PCR amplification differences between DNA extraction kits

**Figure 4:**
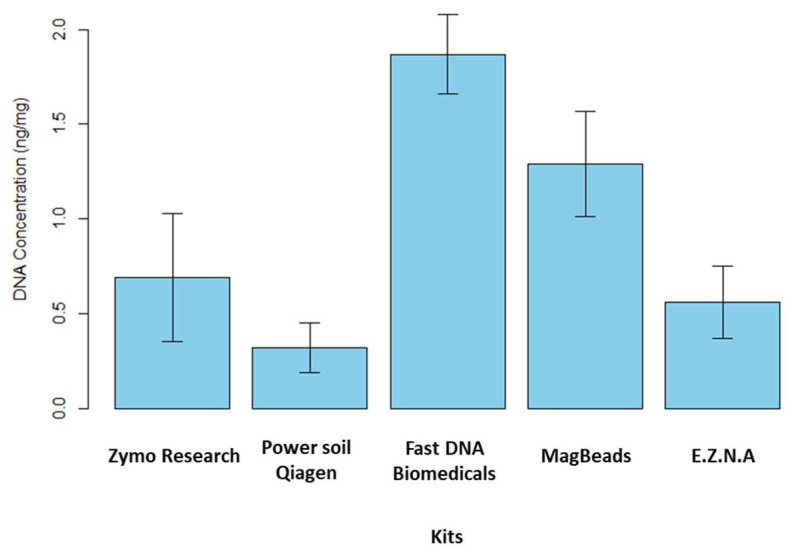
Yield of extracted DNA obtained for the five commercial kits (ng DNA/mg of fresh sediment, 5 replicates). The kits tested in this study are as follows: the Quick-DNA Fecal/Soil Microbe kit (Zymo Research, USA), the DNeasy PowerSoil Pro Qiagen kit (Promega, USA), the FastDNA Spin Kit for Soil, the Magbeads FastDNA kit for Soil (MP Biodemicals, USA) and the E.Z.N.A Soil DNA kit (Omega Biotek, USA).

The study of the microbiome as a link between different trophic levels has been proposed in the One Health paradigm [50]. The reliability of microbiome data depends on the quality of sediment DNA extraction and purification, so that they accurately reflect the composition of sediment microbial communities [51]. Characterizing the environmental microbiome of sediments with great acuity implies being able to identify the locks and/or methodological limits controlling their detection and characterization. In particular, the characterization may depend on the methodology applied to isolate the nucleic acids present in the environmental matrix. Manufacturers have started to release original kits specifically designed for metabarcoding and metagenomics on the market [52]. As previously mentioned in the study carried out by Dopheide et al. [53] on the impact of DNA extraction on soil biodiversity, a consistent DNA extraction method must be applied for comparison between samples. A literature review was carried out to identify the DNA extraction kits described as the most efficient for processing sediment samples, particularly in terms of DNA quantity and quality. We therefore evaluated and compared the efficiency of five commercial DNA extraction kits available on the market in 2024. As a first step, extraction yields were calculated for the five kits tested to determine which one extracted the largest amount of DNA (see Figure 4).

These tests were performed on fresh sediment. In brief, we note that the recovery rate was maximum for FastDNA (MP Biomedicals, East Granby, CT, USA) kit followed by MagBeads FastDNA (MP Biomedicals, East Granby, CT, USA) kit. They extracted c.a. 1.87 ± 0.21 ng·mg^−1^ of sediment and c.a. 1.29 ± 0.28 ng·mg^−1^ of sediment, respectively. The three other extraction kits have a quantity of DNA extracted lower than 1.00 ng·mg^−1^ of sediment. However, it is important to note that the kit manufacturers impose different mass samples for extraction. The FastDNA and MagBeads (MP Biomedicals, East Granby, Connecticut, USA) kits required 500 mg of sample for extraction while, the E.Z.N.A Soil DNA kit (Omega Biotek, Norcross, GA, USA), DNeasy PowerSoil Pro Qiagen kit (Promega, Madison, WI, USA) and Quick-DNA Fecal/Soil Microbe Kit (Zymo Research, Irvine, CA, USA) only required 250 mg of sediment. The A260/A280 ratio was estimated to determine the DNA quality and, in particular, DNA contamination by proteins, phenols or other extraction kit reagents that could absorb at λ = 280 nm while nucleic acids have maximal absorption at λ = 260 nm. The ratio A260/A280 obtained ranked between 1.7 and 2 for all the kits, except Quick-DNA Fecal/Soil Microbe kit (Zymo Research, USA). We noted that for this kit, the error on the ratio value was high. We hypothesize that this result could be due to the low quantity of extracted DNA. Moreover, in order to check the quality of the extracted DNA, PCR amplification tests were carried out on the 16S rRNA gene. Amplification of this gene using specific primers enables us to check the presence or absence of undesirable contaminants. A successful PCR indicates that the isolated DNA is sufficiently pure and undamaged to be successfully amplified, and that no inhibitors or DNA degradation were observed. The 16S rRNA amplicons were analyzed by stained agarose gel electrophoresis, and a band was observed at the expected size of the target 16S rRNA sequence (c.a. 1500 bp). Positive (bacterial DNA isolate) and negative (ultrapure water) controls were run to confirm that amplification reaction was working correctly. All the kits tested here were suitable for PCR amplification In conclusion, our study showed that two DNA extraction kits, FastDNA and MagBeads (MP Biomedicals, USA), stood out in terms of the quantity of DNA extracted, probably due to the higher sediment quantity required by these two kits (500 mg vs. 250 mg for the other kits). On the other hand, in terms of extracted DNA quality, the five kits tested appeared to be relatively equivalent.

- The DNA extraction kit influenced the alpha-diversity and composition of the sediment microbiome

When microbial communities [54] are collected from an ecosystem, it is necessary to evaluate the extent to which a sample reflects the true diversity of the specific niche by evaluating species richness and relative abundance over time and space. Using the ASV table (Table S4), we first calculated rarefaction curves, which correspond to graphical representations of species diversity or amplicon sequence variants (ASVs) in a given sample [55]. These curves show how the number of ASVs increases with the number of sequences, enabling us to estimate the number of ASVs expected in a random sample of individuals taken from our sample collection, and thus to determine whether the sequencing depth was sufficient. Indeed, the number of reads (i.e., the number of genetic sequences) sequenced per sample can vary considerably for purely technical reasons.

**Figure 5:**
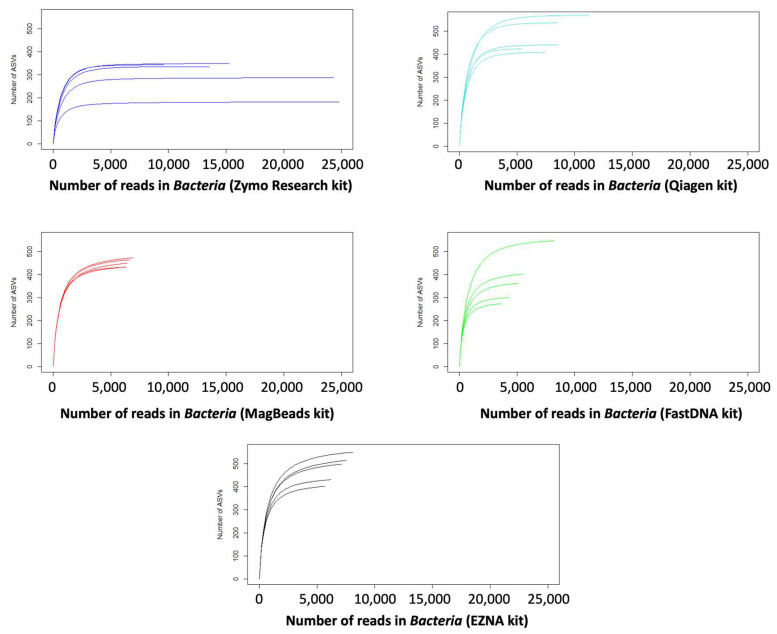
Bacteria domain rarefaction curves indicating the expected taxonomic richness of the samples (number of ASVs) for the five DNA extraction kits. The kits tested in this study are as follows: the Quick-DNA Fecal/Soil Microbe kit (Zymo Research, USA), the DNeasy PowerSoil Pro Qiagen kit (Promega, USA), the FastDNA Spin Kit for Soil, the Magbeads FastDNA kit for Soil (MP Biodemicals, USA), and the E.Z.N.A Soil DNA kit (Omega Biotek, USA).

Since a higher overall number of reads (sequencing depth) increases the chances of detecting rare sequences, richness is positively correlated with sequencing depth. Here, we calculated rarefaction curves to assess whether a fair comparison of richness between microbial communities measured with unequal sequencing depths was possible. Most of the rarefaction curves shown in Figure 5 tended to stabilize with the appearance of a plateau, suggesting that the treatment of our samples reflects the community from which they originate. However, we observe that the plateau was obtained with a slightly smaller number of sequences for the DNeasy PowerSoil Pro Qiagen kit (Promega, USA) and Quick DNA Fecal/Soil Microbe Kit (Zymo Research, USA) compared to the other kits which require a higher number of sequences to reach the plateau on rarefaction metrics. On the other hand, we observed that the DNeasy PowerSoil Pro Qiagen kit (Promega, USA) detected the highest number of ASVs compared to other kits.

**Figure 6:**
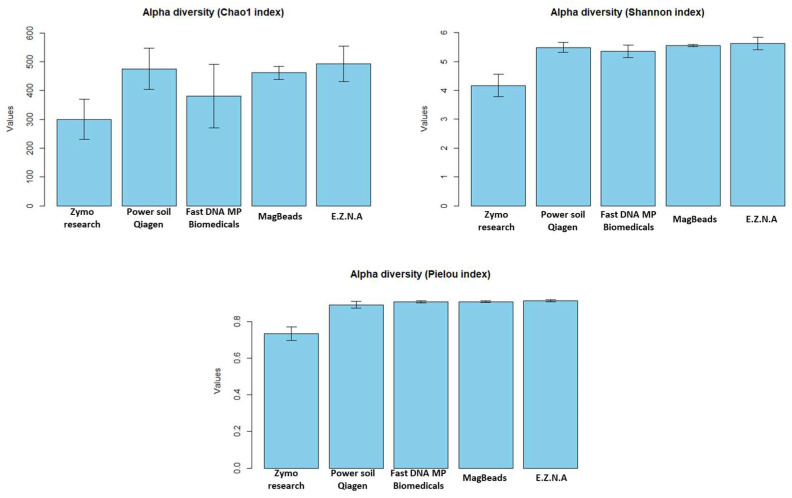
Alpha diversity metrics (Chao1, Shannon and Pielou indexes) of the samples calculated for the five DNA extraction kits. The kits tested in this study were the Quick-DNA Fecal/Soil Microbe kit (Zymo Research, USA), the DNeasy PowerSoil Pro Qiagen kit (Promega, USA), the FastDNA Spin Kit for Soil and the Magbeads FastDNA kit for Soil (MP Biodemicals, USA) and the E.Z.N.A Soil DNA kit (Omega Biotek, USA).

**Figure 7:**
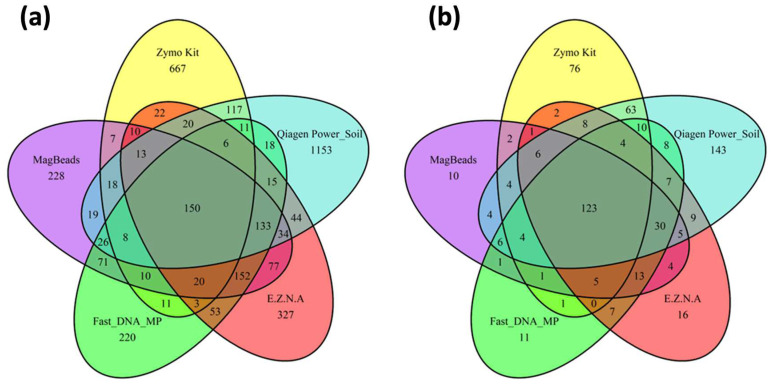
Venn diagrams showing the distribution of (**a**) specific and shared ASVs among kits, (**b**) specific and shared genera among kits. The kits tested in this study were the Quick-DNA Fecal/Soil Microbe kit (Zymo Research, USA), the DNeasy PowerSoil Pro Qiagen kit (Promega, USA), the FastDNA Spin Kit for Soil and the Magbeads FastDNA kit for Soil (MP Biodemicals, USA) and the E.Z.N.A Soil DNA kit (Omega Biotek, USA).

In a second step, we assessed the performance of the five kits in terms of robustness of microbial diversity analysis in order to determine which candidate would be able to provide a metric closest to true sediment diversity for future analyses. Since measures of alpha diversity summarize the structure of an ecological community [56], giving a good overview of ecological parameters in terms of the number of taxonomic groups (richness) and/or the distribution of group abundance (evenness), we first calculated the three most-used diversity indices (Chao 1, Shannon and Pielou) and the number of phyla observed for the five DNA extraction protocols. The estimated alpha diversity indices are shown in Figure 6. We firstly observed that the Power soil Qiagen kit gives the best results compared to the others for the Chao 1 index. A pairwise *t*-test showed that there is a significant difference between Power soil Qiagen and Zymo research kits (*p* value < 0.05). For both Shannon and Pielou indexes, the Power soil Qiagen kit gives similar results to Fast DNA Biomedicals, MagBeads and E.Z.N.A kits (*t*-test *p* value > 0.05). However, it seems to perform better than the Quick-DNA Fecal/Soil Microbe kit (Zymo Research, USA), and this improvement was confirmed by statistical tests (*t* test *p* value = 9.5 × 10^−8^ and 1.4 × 10^−11^ for Shannon and Pielou indexes, respectively). Next, as already proposed by Pearman et al. [26] in a sediment DNA extraction methodology study, Venn diagrams were constructed to assess the distribution of specific and shared ASVs among kits (Figure 7).

The maximum number of ASVs were obtained for the DNeasy PowerSoil Pro Qiagen kit (Promega, USA) and the Quick-DNA Fecal/Soil Microbe Kit (Zymo Research, USA) (*n* = 1785 and *n* = 1093, respectively) with the maximum number of unique ASVs equal to 1153 for DNeasy PowerSoil Pro Qiagen kit (Promega, USA) and 667 for the Quick-DNA Fecal/Soil Microbe Kit (Zymo Research, USA). Considering the bacteria genus, the same two kits had the highest number of unique genus, with 143 unique genera for the PowerSoil Pro Qiagen kit (Promega, USA) and 76 unique genera for the Quick-DNA Fecal/Soil Microbe Kit (Zymo Research, USA). On the other hand, the FastDNA (MP Biomedicals, USA) and MagBeads (MP Biomedicals, USA) were conducted to the lowest number of ASVs values obtained (respectively, *n* = 907 and *n* = 976) with minimum unique ASVs (220 and 228, respectively). These latter kits also exhibited the lowest unique genus level (respectively, *n* = 10 and *n* = 11).

**Figure 8:**
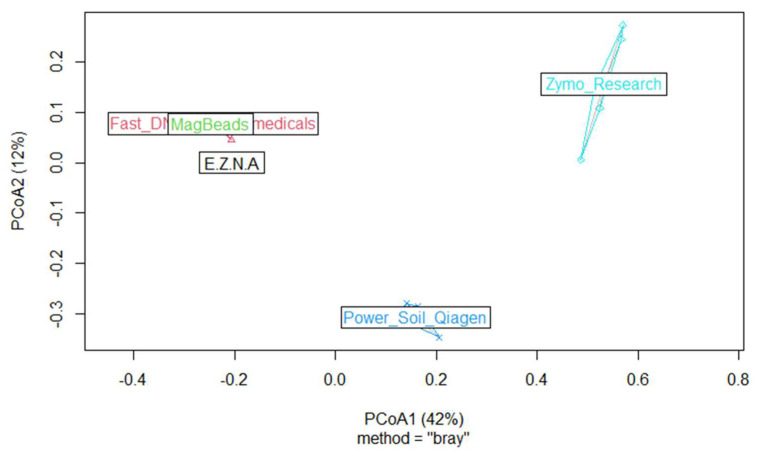
PCoA-bacteria-Data: Multidimensional scaling plots based on the Bray–Curtis distance and reflecting differences in the beta microbial diversity community composition of the five kits.

Finally, in order to describe the composition of the ASVs between samples and to determine whether the choice of DNA extraction kit could influence microbial community composition, we computed one beta diversity metric by using Bray–Curtis dissimilarities (vegdist, {vegan}) algorithm. We processed Principal coordinate analyses (hereafter named PCoA) (pcoa, {vegan}). The PCoA results presented in Figure 8 agreed with the distribution of specific and shared ASVs in the five kits, both at ASVs and genus level; the two kits, DNeasy PowerSoil Pro Qiagen (Promega, USA) and Quick-DNA Fecal/Soil Microbe (Zymo Research, USA), were well separated from the others.

For the molecular microbial ecology methodology development, five commercial kits were compared (the Quick-DNA Fecal/Soil Microbe kit (Zymo Research, USA), the DNeasy PowerSoil Pro Qiagen kit (Promega, USA), the FastDNA Spin Kit for Soil and the Magbeads FastDNA kit for Soil (MP Biodemicals, USA) and the E.Z.N.A Soil DNA kit (Omega Biotek, USA)) in terms of DNA extraction and microbial diversity. Regarding assessment of the alpha-diversity and composition of the sediment microbiome and the number of unique genera, the kit that stood out from the others was the DNeasy PowerSoil Pro Qiagen kit (Promega, USA). The DNA extracted from commercial kits has to be in large amounts and must provide a reproducible representation of the community and diversity in a sample. In this study, we showed that all the kits studied had significantly different microbial community profiles. We identified, from among the five selected commercial DNA extraction and purification kits, the candidate with the best capability to deliver high-quality DNA for downstream molecular applications for sediment microbiome characterization. Although the DNeasy PowerSoil Pro Qiagen kit (Promega, USA) produced lower overall DNA quantities, based on DNA extraction quality (sum of sequences, alpha- and beta-diversity metric indices, the number of unique and shared ASVs and the number of unique and shared genera), we showed that this commercial kit had better DNA quality than the other kits. Consequently, and given our operating conditions, we validated this extraction kit as the most suitable for characterizing the microbial communities occurring in our sediment samples. These results agree with the studies performed by Shi et al. [25] and Pearman et al. [26] using the same commercial kit, which enabled the authors to extract the best-quality DNA from sediment.

## 4. Conclusions

The main objective of this study was to develop an approach combining metabolomics and metabarcoding data. The first part of the manuscript aimed to assess the contamination of the studied lagoon in order to identify, among the organic contaminants present, the candidate molecules likely to be retained as model molecules for future bioreactor experiments. The present work has therefore validated a multidisciplinary approach using chemistry and molecular microbial ecology methodologies to assess (i) the chemical contamination status of the lagoon and (ii) apply metabolomics and 16S metabarcoding methodologies to the sediment. Using semi-targeted methods developed for lagoon contamination, 46 substances belonging to the pharmaceutical, pesticide and hormone steroid groups were detected and 28 were quantified. For metabolomics, sample preparation based on solid/liquid extraction using acetonitrile/methanol (50/50, *v*/*v*) as the extraction solvent showed the best results in terms of both efficiency and reproducibility. Regarding the microbial ecology approach developed in this work, and given the ecological parameters assessed (rarefaction curves, alpha- and beta-diversity and relative abundance), the use of the commercial DNeasy PowerSoil Pro Qiagen Kit (Promega, USA) appears to be the most appropriate choice for characterizing the bacterial diversity of lagoon sediment. This kit provides the most accurate taxonomic view possible of the sedimentary bacterial ecosystem, generated from fresh samples taken from the Canet lagoon. The results obtained in this study have enabled us to validate the methodology for extracting organic compounds, matrix metabolites and DNA from fresh sediments. These results have allowed us to set up the first steps in the workflow of our multi-omics approach. This first step in methodological development was fundamental, as it will enable us to study the fate of contaminants in the lagoon environment and their impact on the microbiome using instrumented bioreactors simulating semi-natural dynamic conditions subjected to organic contaminant exposure scenarios. Regarding the chemical aspect, we now have an effective extraction method associated with chromatographic conditions adapted to our operating conditions. We also identified a commercial DNA extraction kit offering adequate performance for metabarcoding using the 16S rRNA marker.

In terms of perspectives, analytical methodologies targeting polar compounds need to be developed in order to successfully complement the work presented here. Indeed, during the course of this study, we wished to focus on methodological development based on the state of contamination of the lagoon, combined with a metabolic approach targeting mainly semi-polar and apolar compounds. Methodologies adapted to polar substances require more advanced developments involving the use of HILIC-type chromatographic columns combined with the use of NMR. Furthermore, given the high salinity of the Canet lagoon (>9 g/L), which is likely to harbor extremophilic microorganisms, it seems important to study the analysis of the microbial diversity of sediments with the use of primer pairs specifically targeting the *Archaea* phylum in a future work. In this respect, it would be interesting to consider the work of Yang et al. [57], who demonstrated that the *Archaea*-specific primer pair A306F/A713R, specifically targeting the V3–V4 hypervariable region of the 16S rRNA gene, was a very powerful tool for studying microbial diversity in ocean trench sediments.

## Figures and Tables

**Table 1:** Quantitative results of compounds identified in water and sediment samples (*n* = 5 replicates) from the “Canet-St Nazaire” (France, 66) lagoon. Substances tagged with (*) have been quantified using an external calibration method. For the other compounds, an internal calibration was carried out. MLQ = Method Limit of Quantification.

Substances	MLQ Water (ng/L)	Mean Conc Water (ng/L)	CV Water (%)	MLQ Sed. (ng/g)	Mean Conc Sed. (ng/g)	CV Sed. (%)
PHARMACEUTICALS						
Flecainide (Antiarrhythmic)	0.04	35.6	6.15	0.001	0.180	46.5
Oxazepam (Psycholeptic)	0.06	30.6	5.05	0.007	0.088	14.8
Carbamazepine (Psycholeptic)	0.03	22.6	3.30	0.007	0.076	7.21
Fluconazole * (Antimycotic)	0.06	12.1	7.55	0.001	0.030	0
Paracetamol (Analgesic)	1.2	9.15	8.50	0.04	0.092	14.2
Telmisartan * (Hypertensive)	0.01	8.44	20.4	0.02	0.240	47.5
Tiapride (Neuroleptic)	0.002	7.72	6.94	0.001	0.072	22.8
Sulfamethoxazole (Antibiotic)	0.02	7.54	7.36	0.008	nd	-
Valsartan (Hypertensive)	0.06	7.50	5.16	0.1	nd	-
Cetirizine * (Antihistamine/Antiallergic)	0.01	5.34	3.88	0.007	0.042	10.6
Venlafaxine (Antidepressant)	0.05	2.86	24.8	0.001	0.050	0
Disopyramide * (Antiarrhythmic)	0.05	2.76	8.34	0.001	0.054	10.1
Diclofenac (Analgesic)	0.05	1.58	12.1	0.004	0.120	37.3
Amisulpride (Antiarrhythmic)	0.01	1.14	4.80	0.003	0.036	15.2
Citalopram * (Antidepressant)	0.06	0.34	16.1	0.005	0.040	0
PESTICIDES						
Terbuthylazine (Herbicide)	0.02	0.200	0	0.001	0.030	0
Terbuthylazine-2-OH * (by-product)	0.06	47.4	2.78	0.001	0.340	82.1
Terbuthylazine Desethyl hydroxy * (by-product)	0.04	4.40	14.9	0.001	0.028	16.0
Carbendazim (Fungicide)	0.02	12.8	7.85	0.001	26.3	9.55
Atrazine (Herbicide)	0.01	0.660	81.3	0.001	0.660	8.30
Atrazine-2-Hydroxy * (by-product)	0.02	2.98	7.65	0.001	0.0078	44.8
Diuron (Herbicide)	0.05	2.52	3.32	0.01	0.048	17.4
Boscalid (Fungicide)	0.08	2.00	10.0	0.01	0.046	68.0
Propazine-2-Hydroxy * (by-product)	0.02	1.48	3.02	0.001	0.058	39.3
Tebuconazole * (Fungicide)	0.05	1.14	4.80	0.001	0.072	6.21
Imidaclopride (Insecticide)	0.06	0.880	5.08	0.008	nd	-
HORMONAL STEROIDS						
Progesterone * (Progestogen)	0.06	0.0900	11.1	0.005	0.064	34.2
Testosterone * (Androgen)	0.06	nd	nd	0.005	0.042	10.6

**Table 2:** Main endometabolites (metabolites/families) putatively identified in sediments from the Canet-St Nazaire lagoon.

Exp. *m*/*z*	RT (min)	Adduct	Elemental Composition	Putative Compound Class/Family	Putative Compound Identity
96.9599	0.6	[M − H]^−^	H_2_SO_4_	Acid	Sulfuric acid
121.0295	6.8	[M − H]^−^	C_7_H_6_O_2_	Acid	Benzoic acid
268.1043	0.9	[M + H]^+^	C_10_H_13_N_5_O_4_	Nucleoside	Adenosine
287.0890	0.8	[M + FA − H]^−^	C_10_H_14_N_2_O_5_	Nucleoside	Thymidine
327.2913	15.6	[M − H]^−^	C_20_H_40_O_3_	Fatty acyl (fatty acid/fatty ester)	20-hydroxyeicosanoic acid
355.3226	16.5	[M − H]^−^	C_22_H_44_O_3_	Fatty acyl (fatty acid/fatty ester)	2-hydroxydocosanoic acid
369.3378	16.9	[M − H]^−^	C_23_H_46_O_3_	Fatty acyl (fatty acid/fatty ester)	2-hydroxytricosanoic acid
379.2613	11.2/11.3	[M + H]^+^	C_19_H_39_O_5_P	Glycerophospholipid	(isomers)
383.3540	17.4	[M − H]^−^	C_24_H_48_O_3_	Fatty acid	2-hydroxy Lignoceric acid
393.2769	11.8	[M + H]^+^	C_20_H_41_O_5_P	Glycerophospholipid	
496.3394	12.8	[M + H]^+^	C_24_H_50_NO_7_P	Glycerophospholipid (glycerophosphocholine)	1-*O*-palmitoyl-*sn*-glycero-3-phosphocholine
497.2898	12.6	[M − H]^−^	C_23_H_47_O_9_P	Glycerophospholipid	[3-[2,3-dihydroxypropoxy(hydroxy)phosphoryl]oxy-2-hydroxypropyl] heptadecanoate
552.4642	17.8	[M − H]^−^	C_33_H_63_NO_5_	Sphingolipid	
566.4282	7.71	[M + H]^+^	C_30_H_55_N_5_O_5_	Natural cyclopeptide	Clavatustide C
566.4799	18.2	[M − H]^−^	C_34_H_65_NO_5_	Lipid/sphingolipid	
653.5118	17.6	[M − H]^−^	C_37_H_70_N_2_O_7_	Lipopeptide (containing serine)	
753.5292	16.1	[M − H]^−^	C_42_H_70_N_6_O_6_	Peptide	

## Data Availability

No online datasets are available.

## Supplementary Materials

The following supporting information can be downloaded at https://www.mdpi.com/article/10.3390/metabo14080454/s1. Table S1: Labeled internal standards used for extraction; Table S2: Compounds used as quality control for LC-QToF screening; Table S3: Compounds’ MRM transitions used for quantification with H-Class-Xevo-TQ-S; Table S4: ASV table for the 5 DNA extraction kits tested and the 5 replicates, Figure S1. EIC chromatograms for, on the top, putatively identified metabolites in the sediment extract; on the bottom, comparison with blank extraction samples. Chromatograms were selected for the chosen extraction protocol (solid/liquid extraction using acetonitrile/methanol) and obtained with the ESI- ionization mode, Figure S2. EIC chromatograms for, on the top, putatively identified metabolites in the sediment extract; on the bottom, comparison with blank extraction samples. Chromatograms were selected for the chosen extraction protocol (solid/liquid extraction using acetonitrile/methanol) and obtained with the ESI+ ionization mode.
