# Supplementary material for: Validation of the Chemical and Biological Steps Required Implementing an Advanced Multi-Omics Approach for Assessing the Fate and Impact of Contaminants in Lagoon Sediments
Source: Metabolites. 2024 Aug 17;14(8):454. doi: 10.3390/metabo14080454 (PMC11356597; doi:10.3390/metabo14080454)
Supplement: Supplementary file 1 [file metabolites-14-00454-s001.zip › metabolites-3050043-Figures.pdf]

Validation of the Chemical and Biological Steps Required Implementing an Advanced Multi-Omics Approach for Assessing the Fate and Impact of Contaminants in Lagoon Sediments

Anouar Mejait [1], Aurélie Fildier [2], Barbara Giroud [2], Gaëlle Daniele [2], Laure Wiest [2], Delphine Raviglione [1,3,4], Jules Kotarba [1,3], Eve Toulza [5], Triana Ramirez [6,7], Alexia Lanseman [1], Camille Clerissi [1], Emmanuelle Vulliet [2], Christophe Calvayrac [6,7] and Marie-Virginie Salvia [1,3,*]

[1] Centre de Recherches Insulaires et Observatoire de l'Environnement (CRIOBE), 66860 Perpignan, France; anouar.mejait@univ-perp.fr (A.M.); delphine.raviglione@univ-perp.fr (D.R.); jules.kotarba@univ-perp.fr (J.K.); alexia.lanseman@etudiant.univ-perp.fr (A.L.); camille.clerissi@ephe.sorbonne.fr (C.C.);

[2] Institut des Sciences Analytiques UMR 5280, Université Claude Bernard Lyon 1, CNRS, 69100 Villeurbanne, France; aurelie.fildier@isa-lyon.fr (A.F.); barbara.giroud@isa-lyon.fr (B.G.); gaelle.daniele@isa-lyon.fr (G.D.); laure.wiest@isa-lyon.fr (L.W.); emmanuelle.vulliet@isa-lyon.fr (E.V.)

[3] UFR Sciences Exactes et Expérimentales, Université de Perpignan, 66860 Perpignan, France

[4] Plateau MSXM Bio2Mar, Université de Perpignan, 66860 Perpignan, France

[5] IHPE, University Montpellier, CNRS, Ifremer, University Perpignan Via Domitia, 66860 Perpignan, France; eve.toulza@univ-perp.fr

[6] Laboratoire de Biodiversité et Biotechnologies Microbiennes, LBBM, Sorbonne Université, CNRS, F-66650 Banyuls-sur-Mer, France; triana.ramirez@etudiant.univ-perp.fr (T.R.); christophe.calvayrac@univ-perp.fr (C.C.)

[7] Biocapteurs-Analyse-Environnement, Université de Perpignan Via Domitia, 66860 Perpignan, France

* Correspondence: marievirginie.salvia@univ-perp.fr; Tel.: +33-(0)4-30-19-23-09

m/z 121.0295 (benzoic acid)

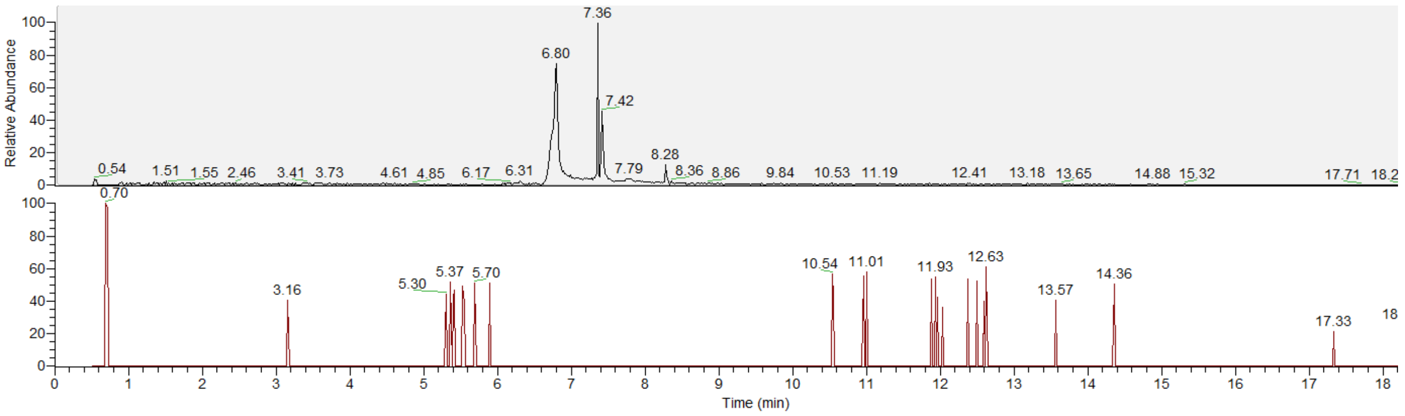

m/z 327.2913 (20-hydroxyeicosanoic acid)

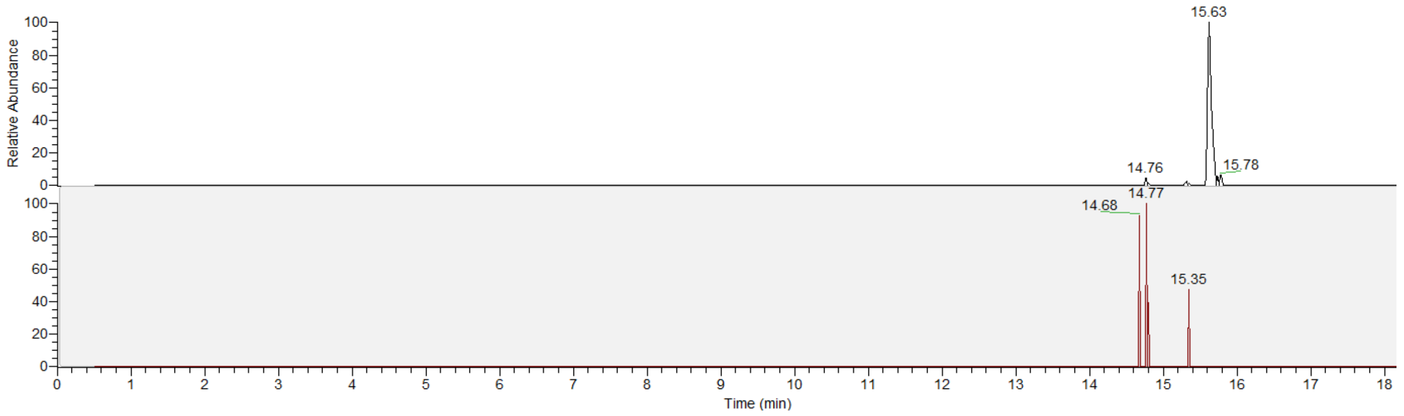

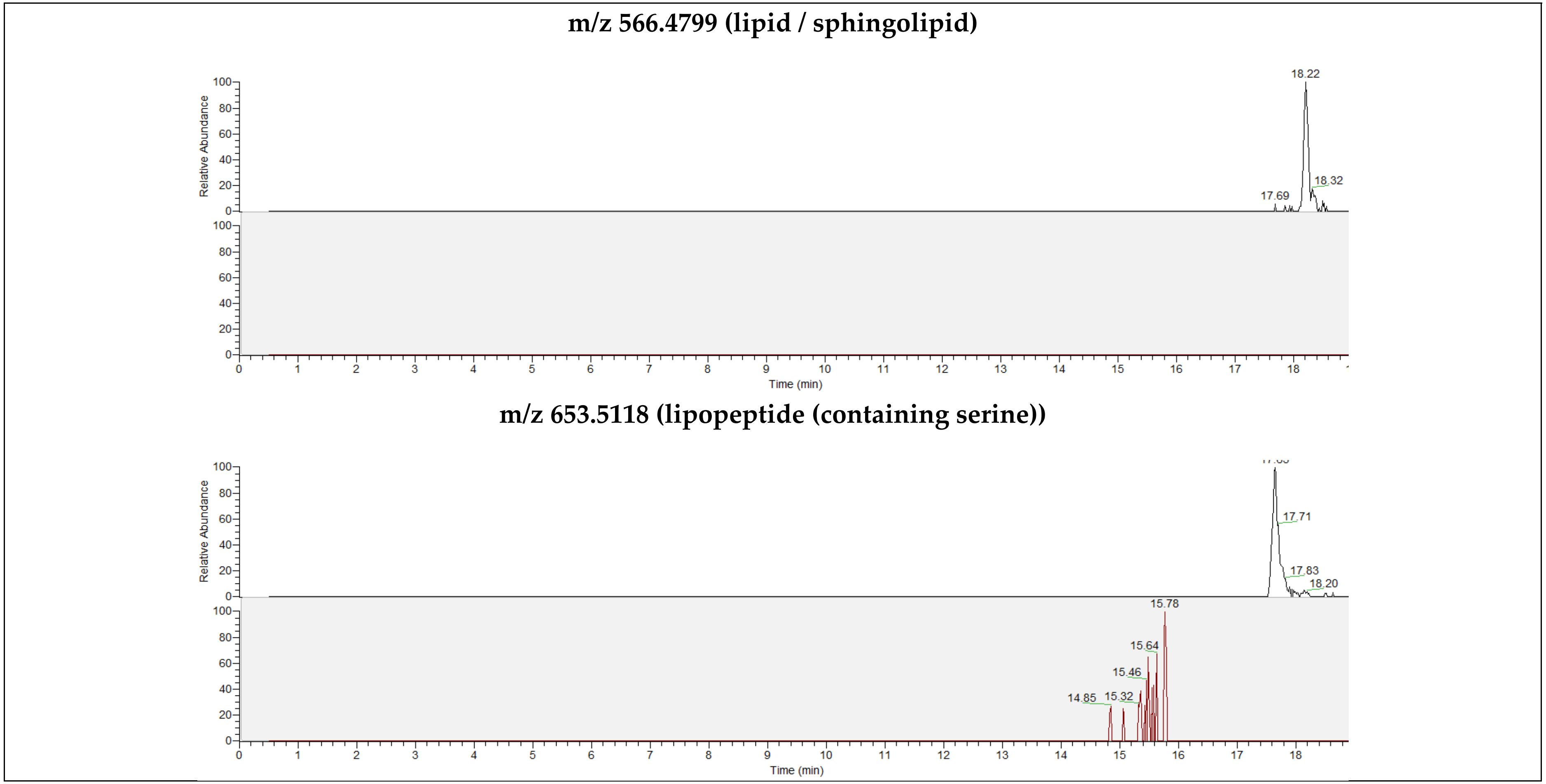

Figure S1: EIC chromatograms for -on the top: putatively identified metabolites in the sediment extract; on the bottom: comparison with blank extraction samples. Chromatograms were selected for the chosen extraction protocol (solid/liquid extraction using acetonitrile/methanol) and obtained with the ESI-ionization mode.

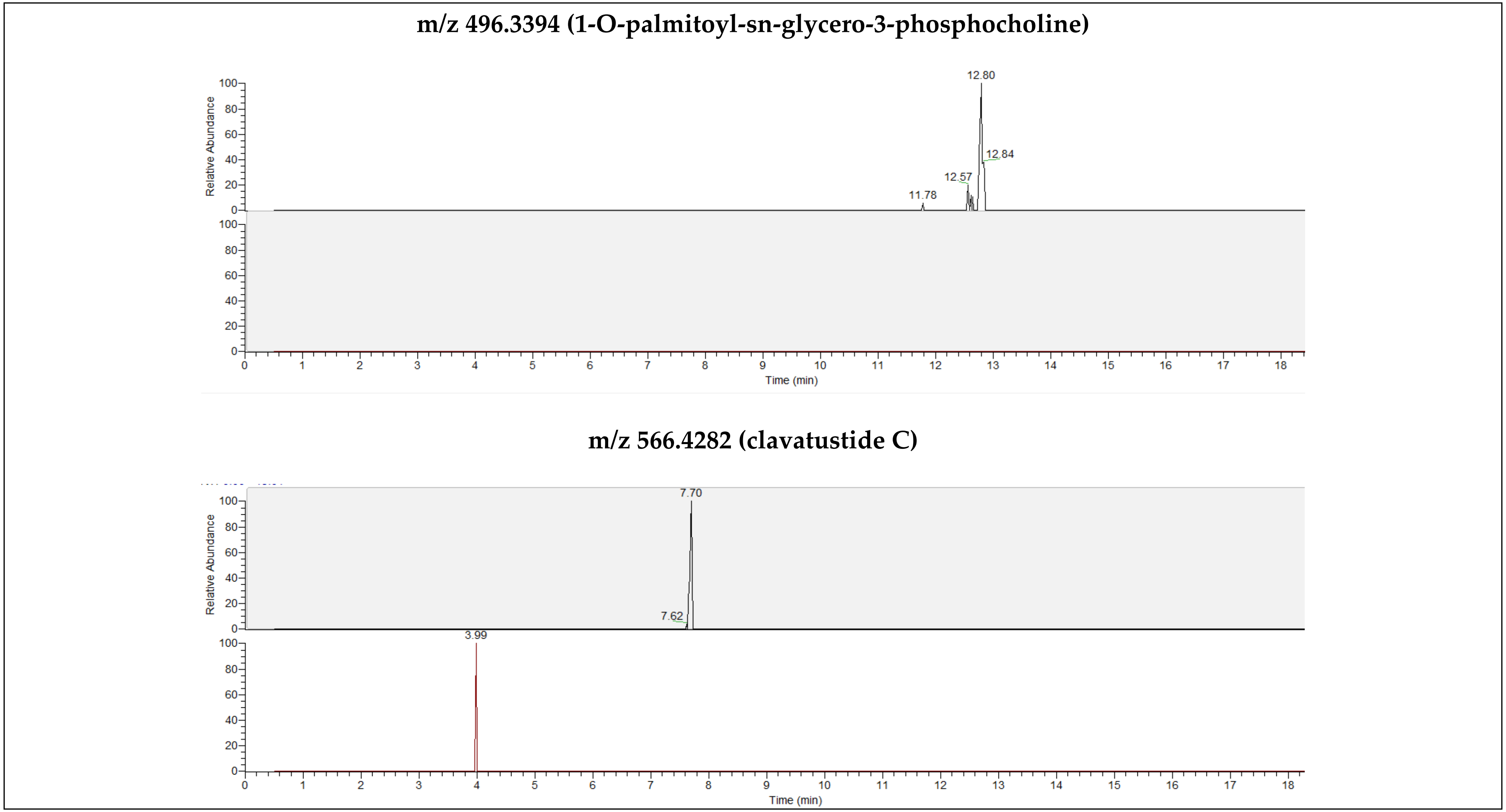

Figure S2: EIC chromatograms for -on the top: putatively identified metabolites in the sediment extract; on the bottom: comparison with blank extraction samples. Chromatograms were selected for the chosen extraction protocol (solid/liquid extraction using acetonitrile/methanol) and obtained with the ESI+ ionization mode.
